# Risk of Covid-19 infection after resection of high grade transitional cell carcinoma with renal impairment

**DOI:** 10.1016/j.ijscr.2021.105924

**Published:** 2021-04-27

**Authors:** Mujalli Mhailan Murshidi, Mohammad Khaled Ihmeidan, Muayyad Mujalli Murshidi, Rand Mujalli (Sheikh Moh'd) Murshidi, Raghad Mujalli Murshidi

**Affiliations:** a Department of Special Surgery/Division of Urology, The University of Jordan, School of Medicine, Queen Rania Street, Amman 11942, Jordan; b Department of Medicine/The Jordainian Royal Medical Services, Queen Rania Street, Amman 11942, Jordan; c Department of Internal Medicine, The University of Jordan, School of Medicine, Queen Rania Street, Amman 11942, Jordan; d Department of Special Surgery/Division of ENT, The University of Jordan, School of Medicine, Queen Rania Street, Amman 11942, Jordan

**Keywords:** Covid-19, Renal impairment, Bladder cancer, Vaccination, Transurethral resection

## Abstract

**Introduction and importance:**

Covid-19 pandemic has had huge impact on health care system and put the health care system under strain, so efforts made to minimize the elective surgeries however some surgeries especially those for high risk malignant tumors cannot be postponed.

The aim of this case report is to highlight the importance of screening cancer patients and those with co-morbidities such as renal impairment for Covid-19 and encouraging them to get vaccinated before undergoing elective surgeries.

**Case presentation:**

We report a case of an 80 year old male patient with renal impairment who developed Covid-19 infection after transurethral resection of high grade transitional cell carcinoma of urinary bladder.

**Clinical discussion:**

Although intra-hospital contagion of Covid-19 is not rare, increased risk of acquiring Covid-19 among cancer patient particularly if they have co-morbidities like renal impairment should be kept in mind and strict protective measures for Covid-19 for those patients should be done before, during and after the procedure.

**Conclusion:**

We theorized that patients with high grade transitional cell carcinoma of urinary bladder should be screened for Covid-19 and get vaccinated before the procedure.

## Highlights

• High grade bladder cancer needs strict and close cystoscopic surveillance.
• Cancer patients are vulnerable to develop severe Covid-19 infection.
• Patients should be screened for Covid-19 before elective procedures.
• Covid-19 vaccination is a priority before elective procedures.

## 1 Introduction

Since the declaration of Covid-19 as global pandemic by world health organization (WHO) on March 11, 2020 [1], Some authorities suggest to stratify urological malignancies into groups to put the surgeries that cannot be deferred in balance of the risk of acquiring infection [2]. The idea behind this stratification was decreasing the risk of Covid-19 transmission among patients, increasing bed availability for Covid-19 patients, relieving the strain on health care system and not to neglect the malignant nature of the disease [2].

## 2 Case presentation

**Fig. 1:**
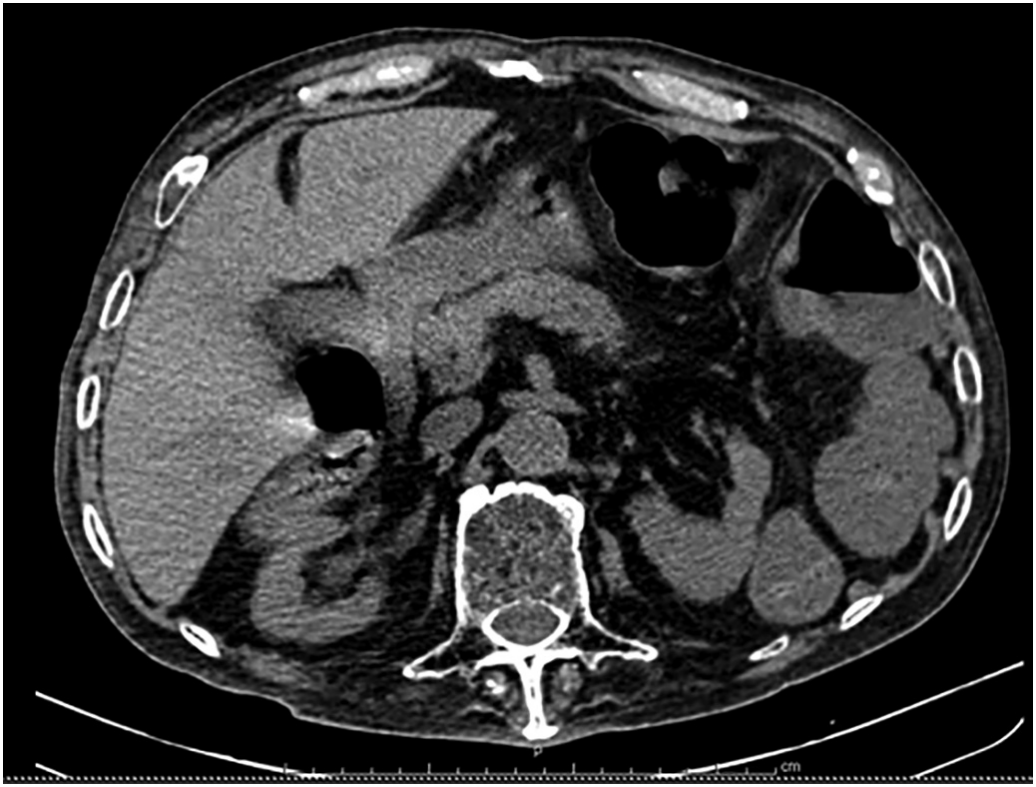
A preoperative non-contrasted computed tomography showing mild right sided hydronephrosis.

An 80 year old male patient with renal impairment, non-smoker, known case of bladder cancer since 2007, missed follow-up since 2014, presented to outpatient clinic complaining of intermittent gross painless hematuria of two months duration, his Covid-19 polymerase chain reaction test was negative on admission, his serum creatinine was elevated (1.9 mg/dl), a non-contrasted computed tomography showed mild right-sided hydroureteronephrosis, bilateral cortical thinning, and diffuse bladder thickening (Fig. 1).

Intraoperatively, a urethral stricture and an enlarged prostate median lobe were seen, multiple growths on bladder neck, posterior wall, both right and left lateral wall more on the right side were seen, too.

After an optical urethrotomy for the stricture, and partial transurethral resection of prostate, an extensive transurethral resection of bladder tumor was done.

The right ureteric orifice could not be seen, a second look cystoscopy was planned in two weeks. The surgery was performed by an experienced senior urologist.

**Fig. 2:**
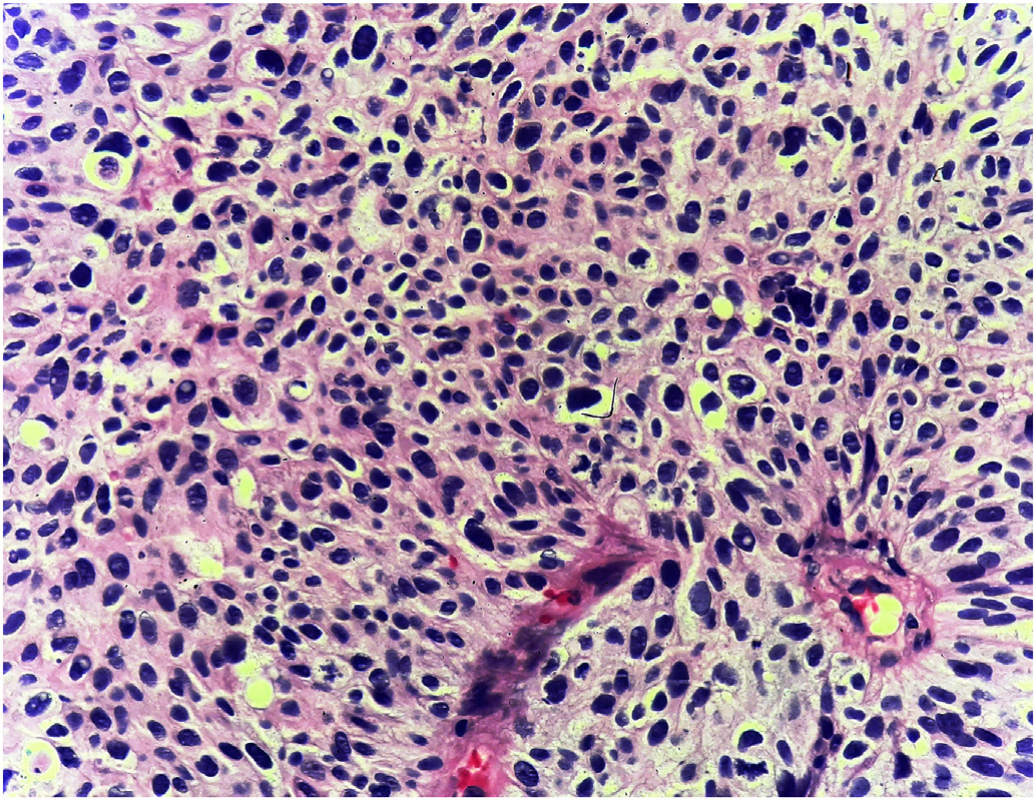
Microscopic picture of the high grade transitional cell carcinoma, hematoxylin and eosin stain. Original magnifications 400×.

The histopathology showed high grade T1 transitional cell carcinoma consistent with T1N0M0 stage (Fig. 2).

On the second post-operative day, a new creatinine was same as baseline (1.9 mg/dl) and the patient was doing well so he discharged to home.

On the third postoperative day, at his home the patient started to feel hotness, chills and rigors, with no cough and no new urinary symptoms.

On the fifth postoperative day, a new Covid-19 test was done at another hospital and it was positive.

On the seventh postoperative day, he started to have shortness of breath, productive cough and generalized weakness, so he presented to another hospital and found to have raised creatinine (8 mg/dl), he was offered admission but he preferred to observe himself at home.

**Fig. 3:**
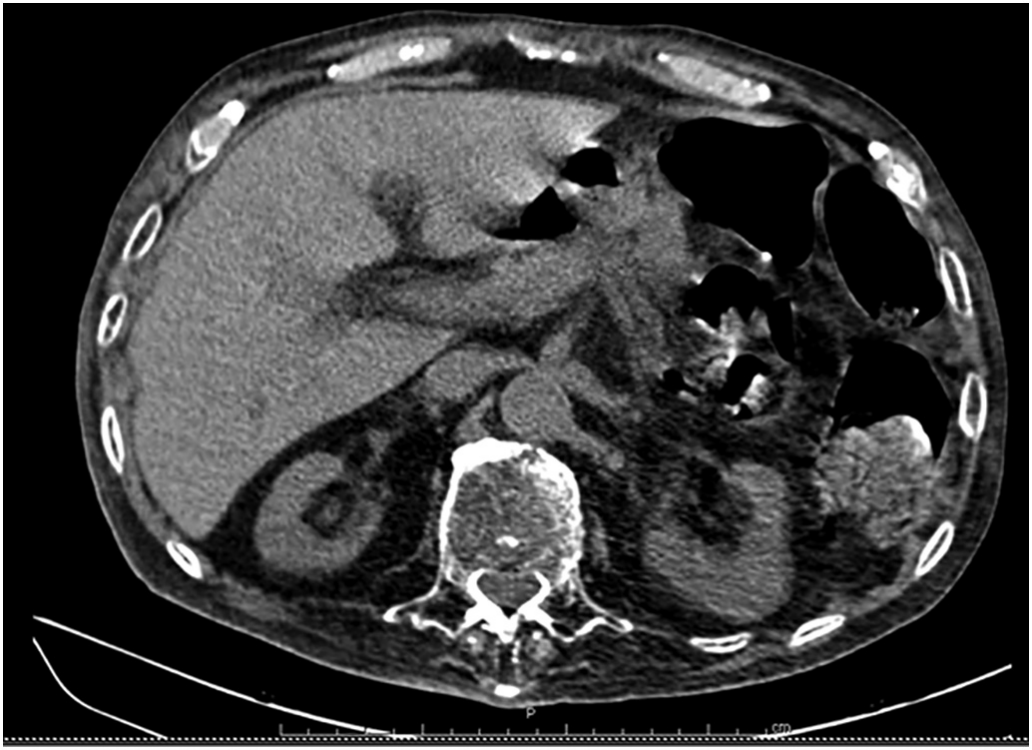
The new non-contrasted computed tomography at the same level showing no new finding compared with the previous one.

On the ninth postoperative day, he came to emergency department at our hospital and found to have serum creatinine of (9 mg/dl), a new computed tomography scan showed no new finding compared to the previous one (Fig. 3).

The nephrologist advised hemodialysis for the patient and to be admitted to intensive care unit, there were no available intensive care unit bed at our hospital, so he was transferred to another hospital where hemodialysis was performed for him.

One month after the procedure, the patient is still hospitalized under medical care due to Covid-19 infection and its complications.

This case has been reported in line with the SCARE 2020 criteria [3].

## 3 Discussion

Bladder cancer is one of tenth most common cancer worldwide, with an estimated 549,000 new cases and 200,000 deaths each year [4].

Covid-19 infection is a serious pandemic with high morbidity and mortality [5]. That affects the hospitalization rate, occupancy and the health strategy to treat patients with elective and semi-elective surgery [6].

It is well known that bladder cancer especially high risk group need strict cystoscopic surveillance to pick the disease up before invasion of the muscularis propria with its serious sequel and increased need for cystectomies and high risk of metastasis [7]. So a balance should be maintained between decreasing non necessary surgeries to those patients and not to leave the bladder tumor to become invasive.

It is believed that patients with malignancy are at increased risk to develop Covid-19 [8]. Other studies confirmed that [9].

The relationship between cancer and increased risk of severe Covid-19 infection may be explained by co morbidities, aging, and innate and cognate immunosuppression [10].

It is believed that Covid-19 virus trigger apoptosis and necroptosis of T lymphocytes and reduce lymphopoietin and interleukin-7 as an immunosuppressive strategy [10,11].

A lot of authors emphasized the interplay between renal impairment and severe Covid-19 infection, and the effect of the baseline kidney impairment on the prognosis and intensive care unit admission rate of Covid-19 patients [12].

## 4 Conclusion

Increased risk of Covid-19 transmission among cancer patients should be kept in mind particularly if they have co-morbidities like renal impairment and good protective measures should be taken. We recommend that patients with bladder cancer should be screened for Covid-19 infection and get vaccinated before the procedure and we adopted this policy in our institution.

## Sources of funding

None.

## Ethical approval

The authors declare that they have no conflict of interest to disclose.

## Consent

Written informed consent was obtained from the patient for publication of this case report and accompanying images. A copy of the written consent is available for review by the Editor-in-Chief of this journal on request.

## Author contribution

Mujalli Murshidi: main surgeon, concept, initiation of the idea, critical analysis, revision of the text and preparation of the final text.

Mohammad Ihmeidan: concept, preparation of the manuscript draft, computer work, and preparation of the final text.

Muayyad Mujalli Murshidi: concept, preparation of the manuscript.

Rand Mujalli (Sheik Moh'd) Murshidi: concept, preparation of the manuscript, and critical analysis.

Raghad Mujalli Murshidi: concept, preparation of the manuscript, and critical analysis.

## Research registration

Not applicable.

## Guarantor

Mujalli Murshidi.

Mohammad Ihmeidan.

## Provenance and peer review

Not commissioned, externally peer-reviewed.

## Declaration of competing interest

The authors declare that they have no conflict of interest to disclose.
